# Multiplexed Cassegrain Reflector Antenna for Simultaneous Generation of Three Orbital Angular Momentum (OAM) Modes

**DOI:** 10.1038/srep27339

**Published:** 2016-06-02

**Authors:** Woo Jin Byun, Kwang Seon Kim, Bong Su Kim, Young Seung Lee, Myung Sun Song, Hyung Do Choi, Yong Heui Cho

**Affiliations:** 1 RF & Antenna Technology Research Section, Electronics and Telecommunications Research Institute (ETRI), Daejeon, 34129, Korea; 2 RF Technology Research Department, Electronics and Telecommunications Research Institute (ETRI), Daejeon, 34129, Korea; 3 School of Information & Communication Convergence Engineering, Mokwon University, Daejeon, 35349, Korea

## Abstract

A multiplexed Cassegrain reflector antenna with a 2 × 2 open-ended rectangular waveguide (OERW) matrix feed and an orbital angular momentum (OAM) mode mux is proposed for the simultaneous generation of three OAM modes (*l* = 0, ±1). The OAM mode mux (OMM) was designed using sequential combinations of quadrature hybrids, crossovers, and phase shifters to multiplex and demultiplex three OAM modes at the same time. The 2 × 2 OERW matrix feed and the OMM were separately measured and their performances were verified according to proposed theories. A near-field antenna measurement for a multiplexed Cassegrain reflector antenna was conducted to obtain the far-field magnitude and phase patterns around polar elevation angle *θ* and azimuthal angle *ϕ*, thus confirming that our antenna can produce three OAM modes simultaneously. We also measured the communication link characteristics of two identical multiplexed antennas. The measurement results show that the channel isolation of three OAM modes is more than 12.7 [dB] and 17 [dB] for fixed and compensated receiver positions, respectively, indicating that the proposed antenna system can be used for independent communication links with the same frequency and polarisation.

It is known that electromagnetic (EM) fields can carry not only the linear momentum described through a Poynting vector but also the angular momentum^1^. The angular momentum consists of the spin angular momentum (SAM) and orbital angular momentum (OAM) which present the polarisation and phase state of the waves, respectively. The concept of OAM modes carried by EM fields is well known in the area of optics^2^, and practical related technologies have also been reported^3^. In contrast to SAM, the theoretical states of OAM are unlimited owing to its unique characteristics of the spiral phase variations. Therefore, novel communication systems based on the OAM modes have a great potential to improve the spectral efficiency and channel capacity of a communication link. However, the areas of application of OAM properties have only recently been translated from optics to radio frequency (RF) technologies^3^,^4^. In the first experimental study on RF OAM modes^5^, EM radio waves encoded into two OAM modes (*l* = 0, +1) on the same operating frequency (2.4–2.48 [GHz]) were separately decoded in the far-field using an interferometer method. Using a planar spiral phase plate, the transmitarray concept was applied to generate arbitrary single and mixed OAM modes^6^. A bidirectional wireless link experiment using three OAM modes (*l* = 0, ±1) was also conducted^7^. Free-space OAM radio was recently proposed based on eight OAM channels (*l* = ±1, ±3) with dual polarisation^8^, where a 32 Gbps millimetre-wave radio link was experimentally demonstrated to verify the possibility of the multiplexing and demultiplexing of eight OAM channels concurrently. However, the distinct difference between OAM modes and a traditional multiple-input multiple-output (MIMO) technique is still questionable^9^,^10^. For instance, the MIMO method could lead to eigenstates identical to the RF OAM modes by allocating uniform circular arrays^9^. The OAM decoding experiment^5^ described above has also aroused controversy regarding its communication efficiency compared to the MIMO method^10^. Because MIMO processing and OAM modes require multiple antenna setups, it is of fundamental and practical importance to investigate simple antenna configurations for the simultaneous generation of different OAM modes without recourse to uniform circular arrays^3^,^4^.

In this paper, we propose a multiplexed Cassegrain reflector antenna with a 2 × 2 matrix feed composed of four open-ended rectangular waveguides (OERWs) to produce three different OAM modes (*l* = 0, ±1) concurrently. To multiplex three OAM modes, an OAM mode mux (OMM) was designed using sequential combinations of quadrature hybrids, crossovers, and phase shifters^11^. An 8 × 8 Butler matrix using microstrip technology to feed a circular array has also been recently considered^12^. In addition, a multiplexed OAM mode antenna composed of circular traveling-wave loop antennas and a ring-focused reflector^13^, which can multiplex and demultiplex two OAM modes (*l* = ±3) concurrently, was proposed. Contrary to circular traveling-wave loop antennas, we show that a Cassegrain reflector antenna with a 2 × 2 OERW matrix feed and an OMM can simultaneously generate three OAM modes (*l* = 0, ±1) and can be extended to a higher OAM mode generation by modifying the *M* × *N* matrix feed and corresponding OMM. For this purpose, the near-field distributions of a multiplexed Cassegrain reflector antenna for each OAM mode were measured and transformed to the far-field in order to check the elevational and azimuthal magnitude and phase variations of three OAM modes. Finally, the OAM mode communication link was tested and verified by sequentially transmitting three OAM modes using a fixed transmitter OAM mode number (*l*_*T*_), and receiving the OAM modes using *l*_*R*_ = *l*_*T*_ (signal reception) and *l*_*R*_ ≠ *l*_*T*_ (isolation), where *l*_*R*_ is the receiver OAM mode number.

## Results

### Multiplexed Cassegrain reflector antenna

**Figure 1:**
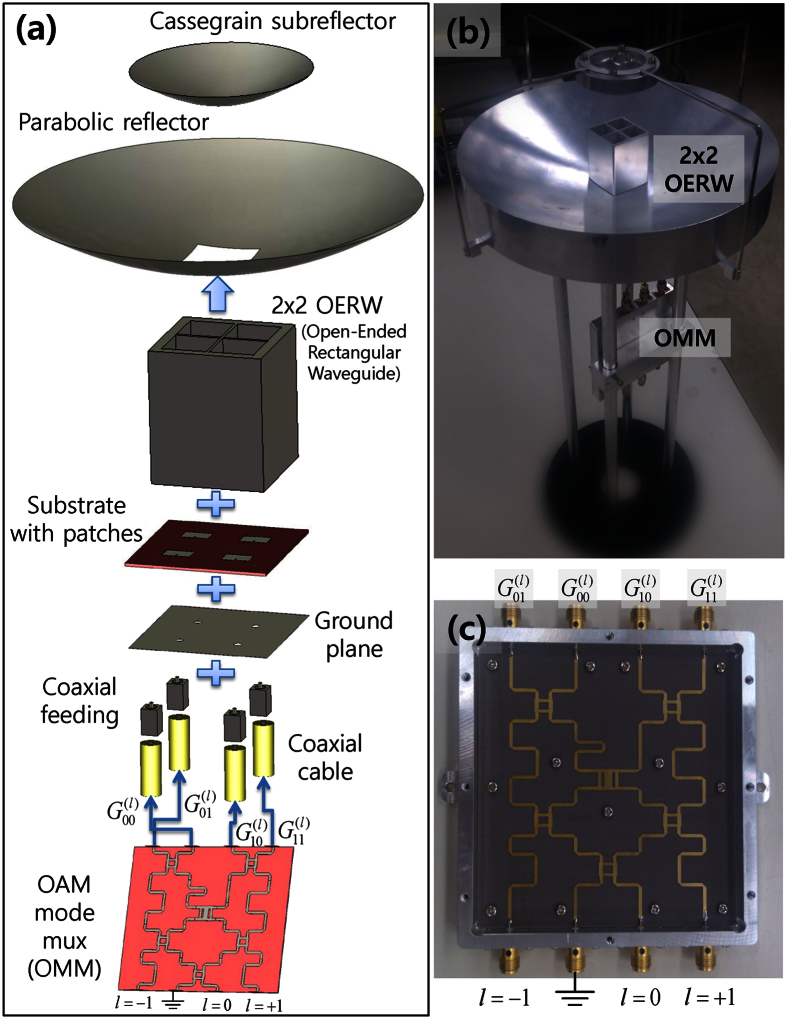
(**a**) Hierarchical diagram of multiplexed Cassegrain reflector antenna composed of parabolic main reflector, Cassegrain subreflector, 2 × 2 OERW matrix feed with substrate and rectangular patches, and OAM mode mux (OMM). (**b**) Image of the proposed antenna. (**c**) Image of the OMM composed of quadrature hybrids, crossovers, and phase shifters using microstrip lines. The inputs of the OMM are used for OAM mode selection (*l* = 0, ±1), and the outputs are directly connected to the 2 × 2 OERW feed.

We propose a multiplexed Cassegrain reflector antenna, illustrated in Fig. 1, composed of a 2 × 2 open-ended rectangular waveguide (OERW) matrix feed, an OAM mode mux (OMM), and a Cassegrain dual-reflector antenna. The 2 × 2 OERW matrix feed, shown in Fig. 1a,b, using four identical OERWs, is an array feed of a multiplexed Cassegrain reflector antenna. Its inputs are fed through an OAM mode mux (OMM)^11^ shown in Fig. 1c, in order to simultaneously generate three OAM modes (*l* = 0, ±1). By combining an aperture electric field of an OERW^14^ in a successive manner and ignoring the electromagnetic coupling among the *M* × *N* OERW feeds, we obtain co-polarised far-field radiation patterns (see the Supplementary Derivation of Equation (1)) as


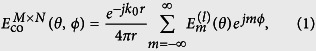


where *k*_0_ = 2*π*/*λ*_0_, *λ*_0_ is free-space wavelength, and 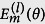 is a modal coefficient of the *m*th-order OAM mode with excitation coefficients 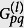, as depicted in Fig. 1c, for the *l*th OAM mode generation. To determine the unknown excitation coefficients 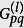, which are outputs of the OMM in Fig. 1c, we assume the condition, 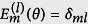 and *M* = *N* = 2, where *δ*_*ml*_ is the Kronecker delta. We then constitute an overdetermined system of simultaneous equations for 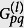 and apply the linear least squares to obtain an optimal solution (see the Supplementary Derivation of Equation (2)) as


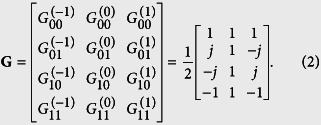


The OAM mode-generating matrix **G** given in Equation (2) becomes an OAM mode mux matrix, which is a key element for the design of an OMM. The components of **G** indicate that we can implement an OMM using conventional passive devices^11^ including quadrature hybrids, crossovers, and phase shifters, as shown in Fig. 1c. Following the standard design procedures of dual-reflector antennas with a Cassegrain subreflector, we designed the multiplexed Cassegrain reflector antenna to generate three OAM modes (*l* = 0, ±1). Differing from ordinary reflector antennas, 2 × 2 OERW matrix feeds are used with a 10 dB beamwidth fixed at 44°. The focus and diameter of the main reflector and subreflector were chosen so as to co-operate the 2 × 2 OERW matrix feed. Based on Equations (1) and (2), we fabricated and measured all components of a multiplexed reflector antenna. An image of a manufactured Cassegrain reflector antenna including the 2 × 2 OERW matrix feed and microstrip OMM is shown in Fig. 1b.

**Figure 2:**
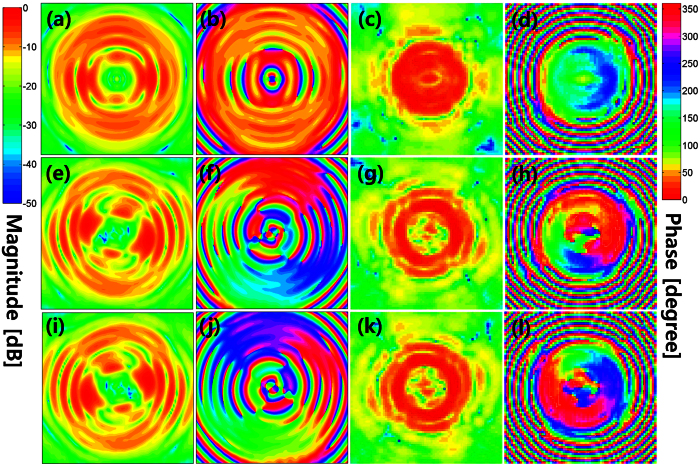
18 [GHz] near-field distributions of multiplexed Cassegrain reflector antenna simulated and measured at *z* = 56 [*mm*] and 183 [*mm*], respectively, when the edge of the main reflector is placed at *z* = 0. (**a**,**b**,**e**,**f**,**i**,**j**) Simulated magnitude and phase of *l* = 0, *l* = +1, and *l* = −1 OAM modes, respectively. (**c**,**d**,**g**,**h**,**k**,**l**) Measured magnitude and phase of *l* = 0, *l* = +1, and *l* = −1 OAM modes, respectively.

**Figure 3:**
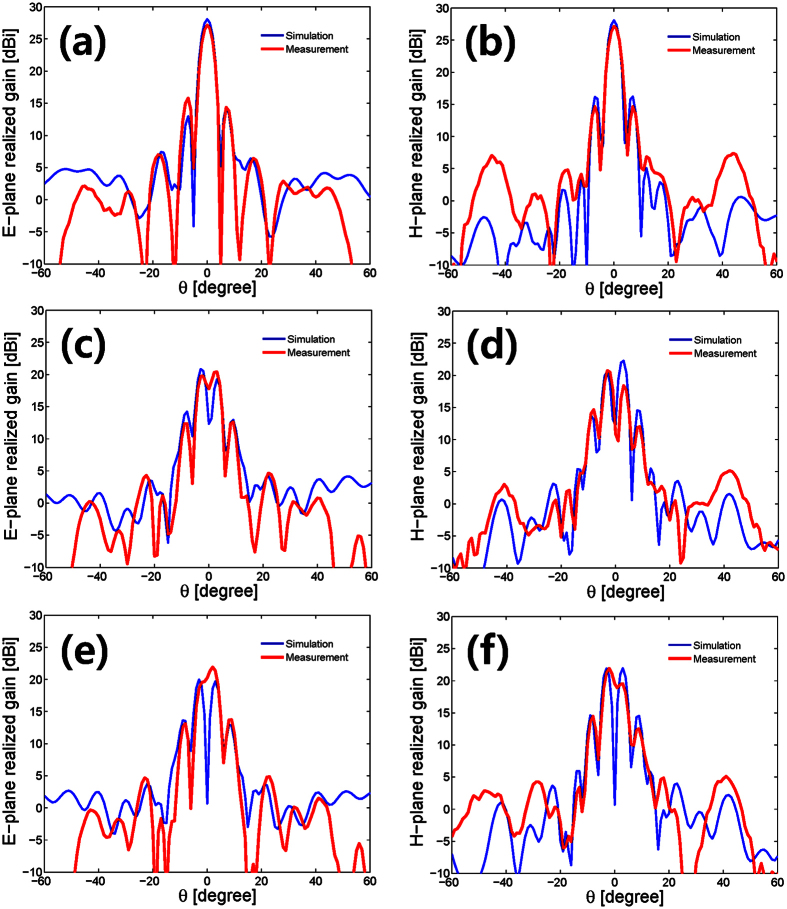
Simulated (blue solid line) and measured (red solid line) far-field radiation patterns of three OAM modes at 18 [GHz]. (**a**,**c**,**e**) E-plane (*ϕ* = 90°) realized antenna gains of *l* = 0, *l* = +1, and *l* = −1 OAM modes, respectively. (**b**,**d**,**f**) H-plane (*ϕ* = 0°) realized antenna gains of *l* = 0, *l* = +1, and *l* = −1 OAM modes, respectively.

**Figure 4:**
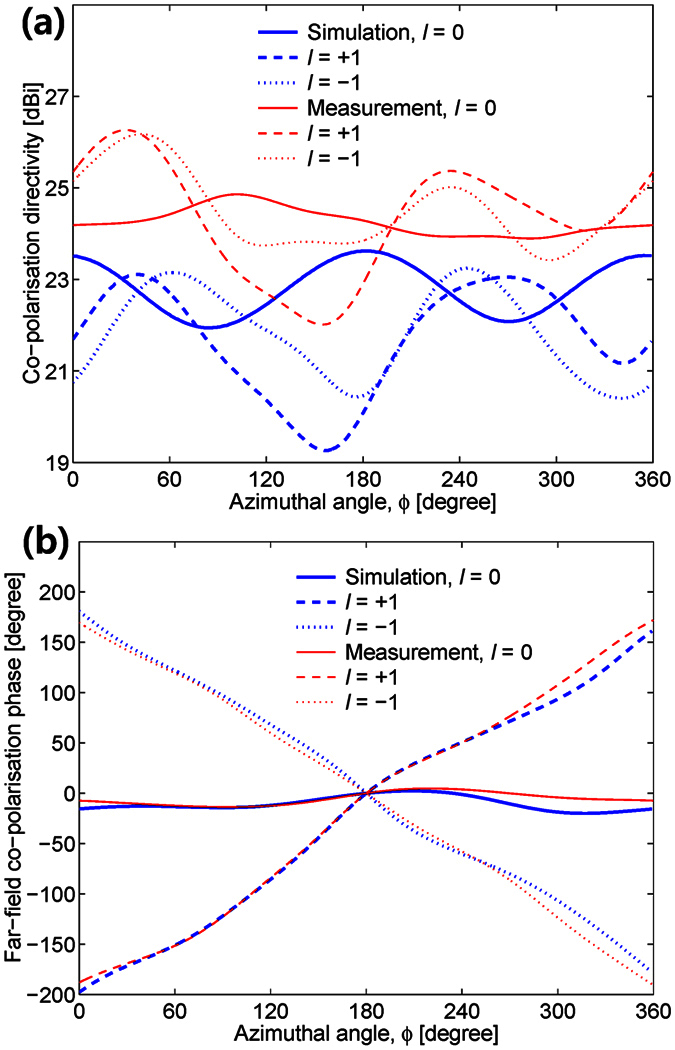
Simulated (blue line) and measured (red line) far-field azimuthal characteristics of *l* = 0 (solid line), *l* = +1 (dashed line), and *l* = −1 (dotted line) OAM modes at 18 [GHz] when *θ* = 3°. (**a**) Co-polarisation directivity. (**b**) Far-field co-polarisation phase.

The proposed antenna was designed and measured using CST Microwave Studio and a near-field measurement system, respectively. The simulated and measured near-field magnitude and phase distributions of the OAM modes for *l* = 0, ±1 are given in Fig. 2. The results indicate that the near-field patterns vary properly according to the OAM states. However, the centre distributions of the magnitude and phase were deteriorated owing to the presence of a Cassegrain subreflector. Using a near-field to far-field transformation, the planar near-field measurement results in Fig. 2 are then converted into the far-field radiation patterns shown in Figs 3 and 4. For the simulation of CST Microwave Studio in Figs 2, 3, 4, we set four inputs 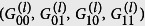 of the 2 × 2 OERW matrix feed using simulated transmission results of the microstrip OMM shown in Fig. 1a (see the Supplementary Simulation and Measurement of OAM Mode Mux). As shown in Fig. 3, the maximum discrepancy at *θ* = 0 for *l* = 0 OAM mode between the simulated and measured antenna gains is 0.89 [dB]. Because gain null points are observed near *θ* = 0, *l* = +1 mode shows a good level of performance. However, *l* = −1 OAM mode shows poor behaviours owing to a deviation of the null points caused by the OMM output phase error. Figure 4 illustrates the far-field azimuthal directivity and phase curves at 18 [GHz] for checking the appropriate generation of the three OAM modes. The differences in directivity between the measured and simulated results in Fig. 4a are maximally 3 [dB]. The far-field phase variations in Fig. 4b also clearly indicate the corresponding OAM states, thus verifying the simultaneous generation of the three OAM modes. These phase-rotating properties of the OAM modes are important factors for telecommunication applications owing to their orthogonal properties. The far-field magnitude and phase characteristics of a multiplexed Cassegrain reflector antenna can be improved by redesigning the OMM using waveguides instead of microstrip lines.

### Link measurement of three OAM mode multiplexed antenna (*l* = 0, ±1)

**Figure 5:**
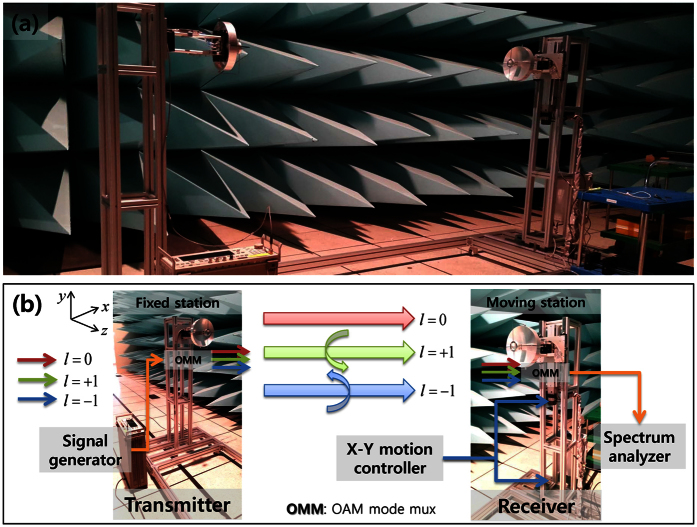
(**a**) Implemented communication link measurement setup for three OAM modes (*l* = 0, ±1). (**b**) Detailed diagram of transmitter (Tx) and receiver (Rx) stations. A signal generator is connected to the Tx and a spectrum analyzer is connected to the Rx. The Rx moves along the *x*- and *y*-axes using an X-Y motion controller.

A communication link composed of three simultaneous OAM modes (*l* = 0, ±1) was measured in terms of the OAM mode reception and isolation. The experiment setup is illustrated in Fig. 5. Because the link of *l* = ±1 is very sensitive to the alignment of the propagation axis (the *z*-axis in Fig. 5b), whereas the *l* = 0 link is insensitive, the multiplexed Cassegrain reflector antennas for the transmitter (Tx) and receiver (Rx) stations in Fig. 5 were separated by 2 [*m*] (see the Supplementary Link Measurement for Different Separation Distance when the separation distances are 1.5 and 2.5 [*m*]) and aligned each other to obtain the maximum power level for the zeroth Tx and Rx OAM modes (*l*_*T*_ = *l*_*R*_ = 0), where *l*_*T*_ and *l*_*R*_ denote the Tx and Rx OAM mode numbers, respectively. We also set the reference Rx position as (*x*_0_, *y*_0_) = (0, 0), which is the position of the maximum power reception of the *l* = 0 OAM mode. For the experiment, the Tx and Rx OAM modes were manually selected by connecting the ports of a signal generator and spectrum analyzer, as shown in Fig. 5b, to the corresponding Tx and Rx OMM ports shown in Figs 1c and 5b.

**Figure 6:**
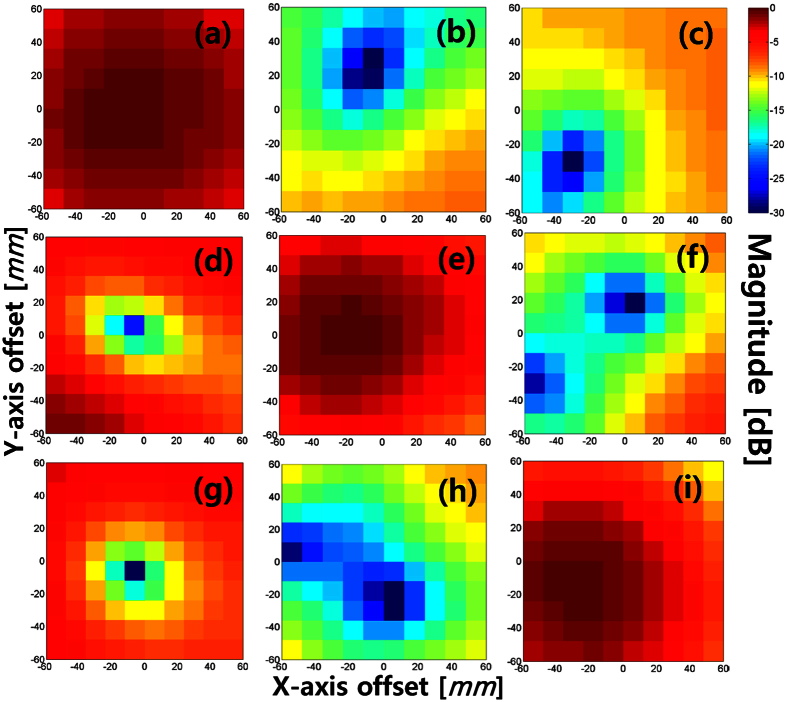
Measured transmission coefficient (*S*_21_) distributions for different transmitter (*l*_*T*_) and receiver (*l*_*R*_) OAM modes when the separation distance between the Tx and Rx is 2 [*m*]. During the measurement, *l*_*T*_ was fixed and *l*_*R*_ was manually chosen by connecting a spectrum analyzer to the corresponding OAM mode port of the OAM mode mux. (**a**–**c**) *l*_*R*_ = 0, *l*_*R*_ = +1, *l*_*R*_ = −1 for *l*_*T*_ = 0, respectively. (**d**–**f**) *l*_*R*_ = 0, *l*_*R*_ = +1, *l*_*R*_ = −1 for *l*_*T*_ = +1, respectively. (**g**–**i**) *l*_*R*_ = 0, *l*_*R*_ = +1, *l*_*R*_ = −1 for *l*_*T*_ = −1, respectively.

The transmission coefficients (*S*_21_) between the Tx and Rx, measured using a signal generator and spectrum analyzer, are shown in Fig. 6. The operating frequency is fixed to 18 [GHz]. The transmission coefficients are normalized with each OAM mode using *l*_*T*_ = *l*_*R*_. The Rx positions (*x*_0_, *y*_0_) for the maximum power reception (*l*_*T*_ = *l*_*R*_ case) were observed to disagree with those of the maximum isolation level (*l*_*T*_ ≠ *l*_*R*_ case). Therefore, we moved the Rx position to the optimal location (*x*_0_, *y*_0_) = (0, −24) [*mm*], where the total signal-to-interference ratio (SIR) is optimized, and is consistently above 11.7 dB for a fixed Rx case. The measured received power and total SIR at (*x*_0_, *y*_0_) = (0, −24) [*mm*] are shown in Table 1. In addition, we moved the Rx position independently for the three transmitting OAM modes, which is provided in Table 2. As a result, each OAM mode has the optimal isolation and total SIR. The related measurements in Table 2 indicate that the isolation level and total SIR can be improved to at least 17 [dB] and 14.9 [dB], respectively. For *l*_*T*_ = −1 and *l*_*R*_ = 0, *S*_21_ is −31 [dB], which indicates a very good level of isolation. Tables 1 and 2, and the Supplementary Link Measurement clearly indicate that the communication link should be precisely aligned within 24 [*mm*] accuracy when the Tx-Rx distance is below 2.5 [*m*].

## Discussion

A multiplexed Cassegrain reflector antenna with a 2 × 2 OERW matrix feed and an orbital angular momentum (OAM) mode mux is proposed for the simultaneous generation of three OAM modes (*l* = 0, ±1). Assuming OAM mode orthogonality, we propose the use of an OAM mode-generating matrix **G**, which is a core element for an OAM mode mux (OMM). Near-field antenna measurements were conducted to illustrate the antenna properties of a multiplexed Cassegrain reflector antenna. The simultaneous generation of OAM modes was confirmed by checking the far-field phase behaviours around the elevational and azimuthal angles. These far-field characteristics for the simultaneous generation of three OAM modes (*l* = 0, ±1) are distinguishable features from the existing literature. Moreover, a communication link measurement revealed that our antenna system is suitable for the simultaneous transmission of three OAM modes with the same frequency and polarisation. According to Tables 1 and 2, and the Supplementary Link Measurement, the total SIR is very sensitive to the Rx position owing to the OAM mode misalignment between the Tx and Rx. Although the physical centers of a multiplexed Cassegrain reflector antenna are the same for three OAM modes, the actual centers are slightly deviated and misaligned by measured errors of the OMM (see the Supplementary Simulation and Measurement of OAM Mode Mux). Detailed discussions on the OAM mode misalignment can be found in terms of an OAM-multiplexed free-space data link^15^. Further investigations will be focused on an improvement in the performance of a multiplexed antenna, such as through the employment of waveguide technology for the design of an OMM, and an extension to a larger sized matrix feed for further generation of higher OAM modes.

## Methods

The components of the 2 × 2 OERW matrix feed, OAM mode mux, and Cassegrain reflector antenna were designed using CST Microwave Studio. According to the simulations and designs, we manufactured the 2 × 2 OERW matrix feed using coaxial feeding, where the OERW (15 × 15 [*mm*^2^]) in Fig. 1a is internally excited using a rectangular patch (7.48 × 4.69 [*mm*^2^]) on a substrate (*ε*_*r*_ = 2.2, thickness = 0.7874 [*mm*]). Four coaxial lines of the 2 × 2 OERW matrix feed beneath the substrate are directly connected to the microstrip OMM. The reflection coefficient of the 2 × 2 OERW matrix feed in Fig. 1a was measured using a network analyzer (Anritsu 37397C) by exciting each OERW with the same magnitude and phase using a 4-way power divider. The simulated and measured reflection coefficients of the matrix feed were below −10 [dB] at 18 [GHz] (see the Supplementary Measurement of 2 × 2 Matrix Feed).

The multiplexed reflector antenna shown in Fig. 1b was mounted onto the planar near-field antenna measurement system. We measured the near-field distributions at 18 [GHz] over square planar grids with a sample spacing of 0.4*λ*_0_, scan area of 462 × 462 [*mm*^2^], and a measurement distance from the subreflector edge to the probe of 8*λ*_0_. For *l* = 0 OAM mode, the measured antenna gain and aperture efficiency at 18 [GHz] are 27.7 [dBi] and 30%, respectively. Note that insertion losses owing to the use of a microstrip OMM, an isolator, and coaxial cables were compensated during the CST simulation. The measurement results are shown in Fig. 3.

To conduct the communication link measurement, we set up a signal generator (Agilent E8267D) for the transmitter (Tx) and a spectrum analyzer (Agilent 8563EC) for the receiver (Rx), where the Tx was fixed to the metal frame and the Rx was moved using an X-Y motion controller (Autonics). The output power of the signal generator was 12 [dBm] at 18 [GHz]. In addition, the Tx and Rx antennas were elevated 1.5 [*m*] from the ground, and the separation distance between the Tx and Rx was 2 [*m*].

## Additional Information

**How to cite this article**: Byun, W. J. *et al*. Multiplexed Cassegrain Reflector Antenna for Simultaneous Generation of Three Orbital Angular Momentum (OAM) Modes. *Sci. Rep.* **6**, 27339; doi: 10.1038/srep27339 (2016).

## Supplementary Material

Supplementary Information

## Figures and Tables

**Table 1:** Optimal transmission coefficients (*S*_21_) and total signal-to-interference ratio (SIR) when OAM mode number of the transmitter (Tx) is fixed to *l*_*T*_, the receiver (Rx) position is anchored to (*x*_0_, *y*_0_) = (0, −24) [*mm*], and the separation distance between the Tx and Rx is 2 [*m*].

**Tx**	**Fixed Rx at 18 [GHz]**		
***S***_**21**_	***l***_***R***_** = 0**	***l***_***R***_** = +1**	***l***_***R***_** = −1**
*l*_*T*_ = 0	0	−21.8	−14.2
*l*_*T*_ = +1	−18.8	0	−19
*l*_*T*_ = −1	−12.7	−21.3	0
Total SIR	11.7	18.6	12.9

**Table 2:** Best *S*_21_ and total SIR when the Rx position for a fixed *l*_*T*_ is moved slightly to (*x*_0_, *y*_0_).

**Tx**	**Compensated Rx at 18 [GHz]**			(***x***_**0**_, ***y***_**0**_)
***S***_**21**_	***l***_***R***_** = 0**	***l***_***R***_** = +1**	***l***_***R***_** = −1**	[***mm***]
*l*_*T*_ = 0	0	−17.8	−17	(−12, −24)
*l*_*T*_ = +1	−18.8	0	−19	(0, −24)
*l*_*T*_ = −1	−31	−23.3	0	(−12, −12)
Total SIR	18.6	16.8	14.9	–

The other parameters are the same as those in Table 1.
