# Supplementary material for: Multiplexed Cassegrain Reflector Antenna for Simultaneous Generation of Three Orbital Angular Momentum (OAM) Modes
Source: Sci Rep. 2016 Jun 2;6:27339. doi: 10.1038/srep27339 (PMC4890295; doi:10.1038/srep27339)
Supplement: Supplementary Information [file srep27339-s1.pdf]

Multiplexed Cassegrain Reflector Antenna for Simultaneous Generation of Three Orbital Angular Momentum (OAM) Modes

Woo Jin Byun*, Kwang Seon Kim*, Bong Su Kim*, Young Seung Lee*, Myung Sun Song*, Hyung Do Choi†, and Yong Heui Cho‡

*RF & Antenna Technology Research Section, Electronics and Telecommunications Research Institute (ETRI), Daejeon, 34129, Korea

†RF Technology Research Department, Electronics and Telecommunications Research Institute (ETRI), Daejeon, 34129, Korea

‡School of Information & Communication Convergence Engineering, Mokwon University, Daejeon, 35349, Korea

e-mail: yongheui.cho@gmail.com

I. Supplementary Derivation of Equation (1)

A multiplexed Cassegrain reflector antenna is composed of a 2×2 matrix feed, an OAM mode mux (OMM), and a Cassegrain dual-reflector antenna. We will present the detailed theoretical derivations on how to produce an OAM mode-generating matrix from a generalized $M \times N$ matrix feed. An $e^{j\omega t}$ time convention is assumed and omitted throughout. Fig. 1 illustrates the geometry of a 2×2 matrix feed. A 2×2 matrix feed is an array feed of a multiplexed Cassegrain reflector antenna. Its inputs are fed through an OAM mode mux (OMM) to generate three OAM modes ($l = 0, \pm 1$). The 2×2 matrix feed in Fig. 1 has four identical open-ended rectangular waveguides (OERWs) with the same width and height. Since the far-field radiation patterns of a single OERW are already formulated in [1], we can represent the electric fields of a generalized $M \times N$ array of OERWs. By combining an aperture E-field of an OERW [1] in a successive manner and ignoring the electromagnetic coupling among OERWs, we can approximately define that of a $M \times N$ matrix feed as

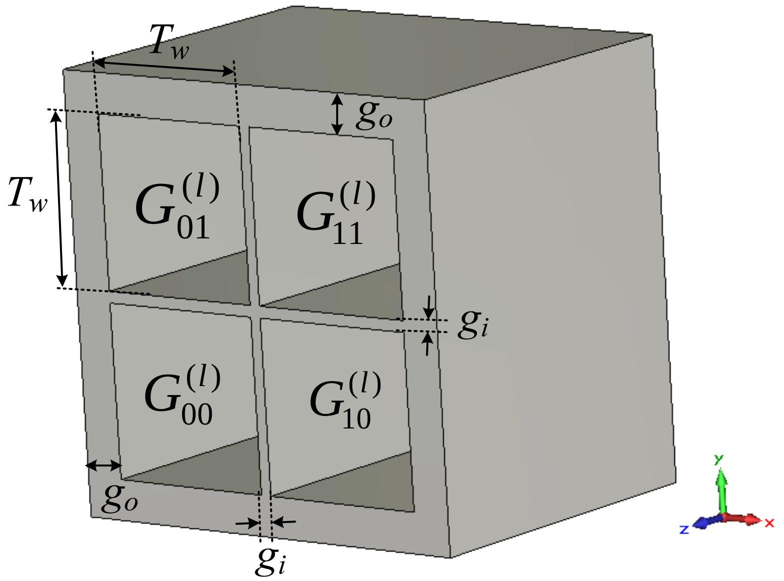

Fig. 1. Geometry of a 2×2 matrix feed composed of four open-ended rectangular waveguides (OERWs) with excitation coefficients $G_{pq}^{(l)}$.

$$\bar{E}_y^{M\times N}(x,y) \approx \sum_{p=0}^{M-1}\sum_{q=0}^{N-1} G_{pq}^{(l)} \times \bar{E}_y^{W}\Big[x + [p-(M-1)/2]T, y + [q-(N-1)/2]T\Big], \tag{1}$$

where M and N are the numbers of OERWs for the x- and y-axes, respectively, $G_{pq}^{(l)}$ is an excitation coefficient for the OERW placed at the center position $(x_c, y_c) = (pT - T/2, qT - T/2)$ to generate the OAM mode of $m = l$, $l = 0$ or $\pm\lfloor (MN-1)/2 \rfloor$, $\lfloor \cdot \rfloor$ is a floor function, $T = T_w + g_i$, and $\bar{E}_y^W(x,y)$ is a y-directed E-field on the OERW aperture [1] for $-T_w/2 < (x,y) < T_w/2$ and $z = 0$. Note that we assume the position $z = 0$ indicates the opening tip of an OERW. Based on the Ludwig's third definition of cross-polarization [2], we asymptotically compute the E-fields of reference $[E_{\text{co}}^{M\times N}(\theta,\phi)]$ and cross

$[E_{\text{cx}}^{M\times N}(\theta,\phi)]$ ones in the far-field from (1), respectively, as

$$E_{\text{co}}^{M\times N}(\theta,\phi) \sim \frac{e^{-jk_0 r}}{k_0 r}\left[E_E(\theta)\sin^2\phi + E_H(\theta)\cos^2\phi\right] \times \sum_{m=-\infty}^{\infty} F_m^{(l)}(\theta)e^{jm\phi} \quad (2)$$

$$E_{\text{cx}}^{M\times N}(\theta,\phi) \sim \frac{e^{-jk_0 r}}{k_0 r}\left[E_E(\theta) - E_H(\theta)\right]\sin\phi\cos\phi \times \sum_{m=-\infty}^{\infty} F_m^{(l)}(\theta)e^{jm\phi}\ , \quad (3)$$

where $k_0 = 2\pi/\lambda_0$, λ_0 is free-space wavelength, the normalized E- and H-plane radiation patterns of an OERW, $E_E(\theta)$ and $E_H(\theta)$, are given in [1],

$$F_m^{(l)}(\theta) = j^m \sum_{p=0}^{M-1}\sum_{q=0}^{N-1} G_{pq}^{(l)} e^{-jm\psi_{pq}} J_m\left[k_0\rho_{pq}\sin\theta\right] \quad (4)$$

$$\rho_{pq} = T\sqrt{[p-(M-1)/2]^2 + [q-(N-1)/2]^2}\ , \quad (5)$$

$J_m(\cdot)$ is the mth-order Bessel function of the first kind, and $\psi_{pq} = \tan^{-1}[(2q{-}N+1)/(2p{-}M+1)]$. Utilizing (2) and the OAM modal expansion, we can also define the co-polarized E-field as

$$E_{\text{co}}^{M\times N}(\theta,\phi) = \frac{e^{-jk_0 r}}{4\pi r}\sum_{m=-\infty}^{\infty} E_m^{(l)}(\theta)e^{jm\phi}\ , \quad (6)$$

where $E_m^{(l)}(\theta)$ is a modal coefficient of the mth-order OAM mode with excitation coefficients $G_{pq}^{(l)}$ and it is related to $F_m^{(l)}(\theta)$ as

$$\frac{E_m^{(l)}(\theta)}{\lambda_0} = \left[E_E(\theta) + E_H(\theta)\right]F_m^{(l)}(\theta) + \frac{E_H(\theta) - E_E(\theta)}{2}\left[F_{m-2}^{(l)}(\theta) + F_{m+2}^{(l)}(\theta)\right]\ . \quad (7)$$

When $E_E(\theta) = E_H(\theta)$, (6) simply reduces to

$$E_{\text{co}}^{M\times N}(\theta,\phi) = \frac{\lambda_0 e^{-jk_0 r}}{2\pi r}E_E(\theta)\sum_{m=-\infty}^{\infty} F_m^{(l)}(\theta)e^{jm\phi}\ . \quad (8)$$

The special condition $E_E(\theta) = E_H(\theta)$ means that the E-plane radiation pattern is identical to the H-plane pattern.

II. Supplementary Derivation of Equation (2)

By assuming the condition, $E_m^{(l)}(\theta) = \delta_{ml}$ and $M = N = 2$, to determine unknown excitation coefficients $G_{pq}^{(l)}$ for Fig. 1, we constitute an overdetermined system of simultaneous equations for $G_{pq}^{(l)}$ from the relation (7). When l and θ are fixed, extracting $G_{pq}^{(l)}$ from (4) and (7), and equating it with δ_{ml} yields the mth simultaneous equation as

$$\sum_{p=0}^{1}\sum_{q=0}^{1} M_{m,2p+q} G_{pq}^{(l)} \;=\; \delta_{ml} \;, \tag{9}$$

where $M_{m,2p+q}$ is a coefficient of $G_{pq}^{(l)}$. Therefore, the final results of (9) can be written in terms of matrix form as

$$\mathbf{M}\mathbf{G}^{(l)} = \mathbf{I}^{(l)} \;\Leftrightarrow\; \left[M_{m,2p+q}\right] \begin{bmatrix} G_{00}^{(l)} \\ G_{01}^{(l)} \\ G_{10}^{(l)} \\ G_{11}^{(l)} \end{bmatrix} = \left[\delta_{ml}\right] \;, \tag{10}$$

where δ_{ml} is the Kronecker delta, $-2 \leq m \leq 2$, $-1 \leq l \leq 1$, and $2p+q$ means the column position of $\mathbf{M}$ for $G_{pq}^{(l)}$. To optimally determine $\mathbf{G}^{(l)}$, we apply the linear least squares [3] to (10) and obtain the best solution as

$$\mathbf{G}^{(l)} \;=\; \left(\mathbf{M}^T\mathbf{M}\right)^{-1}\mathbf{M}^T\mathbf{I}^{(l)} \;, \tag{11}$$

where $(\cdot)^T$ is a transpose of $(\cdot)$. Using (11), we calculated $G_{pq}^{(l)}$ for $l = 0, \pm 1$ with the parameters: f = 18 [GHz], T_w = 15 [mm], g_i = 1 [mm], g_o = 3.5 [mm], and θ = 15°. Collecting $\mathbf{G}^{(l)}$ in (11) for all l, an OAM mode-generating matrix $\mathbf{G}$ is, therefore, given by

$$\begin{aligned} \mathbf{G} \;&=\; \left[\mathbf{G}^{(-1)} \;\; \mathbf{G}^{(0)} \;\; \mathbf{G}^{(1)}\right] \\ &=\; \frac{1}{2}\begin{bmatrix} 1 & 1 & 1 \\ 0.998\angle 90.8^\circ & 1 & 1.002\angle{-90.8^\circ} \\ 0.998\angle{-89.2^\circ} & 1 & 1.002\angle 89.2^\circ \\ -1 & 1 & -1 \end{bmatrix} \;. \end{aligned} \tag{12}$$

Although the elements in (12) are the best excitation coefficients to satisfy $E_m^{(l)}(\theta) = \delta_{ml}$ in the sense of least squares, it is practically difficult to generate $\mathbf{G}$ in (12) with ordinary quadrature hybrids and phase shifters. Thus, we modify (12) to the matrix suitable for an OAM mode mux (OMM) as

$$\mathbf{G} \;=\; \frac{1}{2}\begin{bmatrix} 1 & 1 & 1 \\ j & 1 & -j \\ -j & 1 & j \\ -1 & 1 & -1 \end{bmatrix} \;. \tag{13}$$

The OAM mode-generating matrix $\mathbf{G}$ given in (13) will become a mode mux matrix that is a key element for the design of an OMM. It is interesting that column vectors of $\mathbf{G}$ in (13) are orthonormal each other, thus yielding the identity condition as

$$\mathbf{G}^\dagger\mathbf{G} = \mathbf{I} \;, \tag{14}$$

where $(\cdot)^\dagger$ is a conjugate transpose of $(\cdot)$ and $\mathbf{I}$ is an identity matrix. Fig. 2 shows the simulated radiation patterns of a 2×2 matrix feed versus azimuthal angle ϕ with fixed polar angle $\theta = 15^\circ$. Although the magnitude patterns of $l = 0, \pm 1$ have some ripples around ϕ, the co-polarization phase behaviors vary as they should be. In Fig. 2, we can also observe that our closed-form formulation (2) agrees well with the CST simulation results both for magnitude and phase. The OAM mode-selection behaviors are illustrated

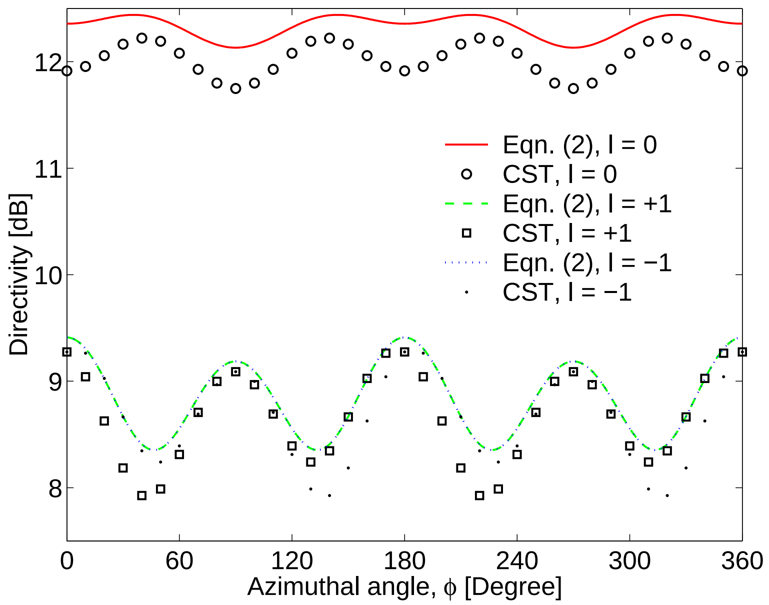

(a)

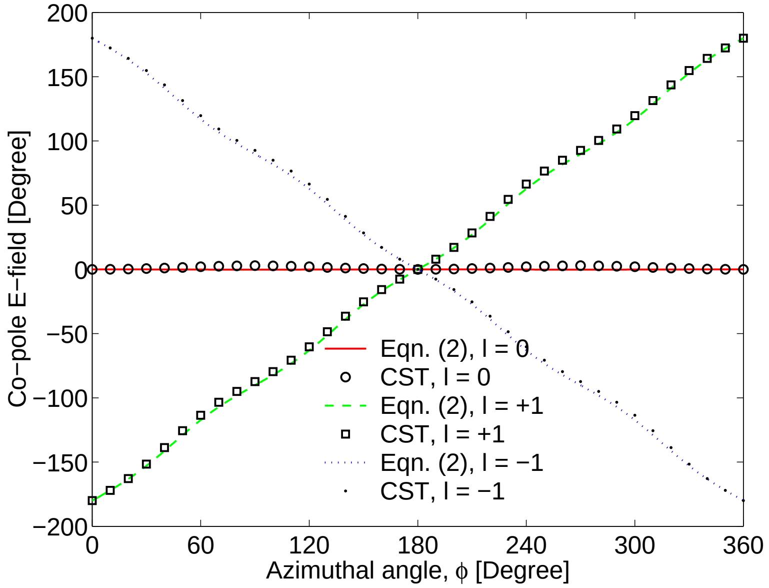

(b)

Fig. 2. Simulated radiation characteristics of a 2×2 matrix feed composed of four OERWs with f = 18 [GHz], T_w = 15 [mm], g_i = 1 [mm], g_o = 3.5 [mm], θ = 15°, and $\mathbf{G}$ in (13) : (a) directivity, (b) co-polarization phase.

in Fig. 3 in terms of normalized field coefficients $20\log_{10}\left|E_m^{(l)}(\theta)/E_l^{(l)}(\theta)\right|$ at $\theta = 15°$. For instance, in the case of $l = -1$, the excitation vector $\mathbf{G}^{(-1)}$ successfully suppresses spurious OAM modes, $l = 0$ and $+1$, below 50 [dB]. Therefore, Fig. 3 clearly indicates that an OAM mode-generating matrix $\mathbf{G}$ in (13) is well defined to produce three OAM modes independently.

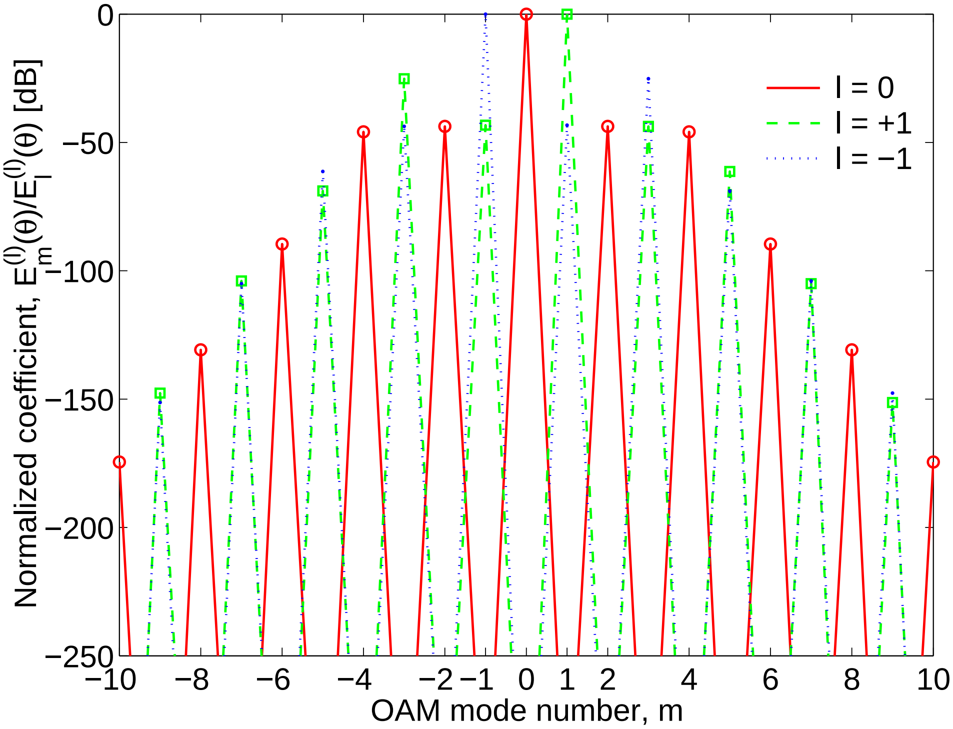

Fig. 3. Behaviors of normalized field coefficients $20\log_{10}\left|E_m^{(l)}(\theta)/E_l^{(l)}(\theta)\right|$ versus OAM mode number m with the same parameters in Fig. 2.

III. Supplementary Simulation and Measurement of OAM Mode Mux

Input	$G_{01}^{(l)}$	$G_{00}^{(l)}$	$G_{10}^{(l)}$	$G_{11}^{(l)}$
$l = 0$ (CST)	$0.371\angle$-179°	$0.330\angle 180^\circ$	$0.355\angle 180^\circ$	$0.310\angle 178^\circ$
(Meas.)	$0.364\angle$-10.6°	$0.274\angle$-6.45°	$0.305\angle$-15.4°	$0.255\angle$-2.99°
$l = +1$ (CST)	$0.339\angle 39.3^\circ$	$0.346\angle 128^\circ$	$0.355\angle$-138°	$0.382\angle$-48.5°
(Meas.)	$0.276\angle$-161°	$0.355\angle$-74.2°	$0.242\angle 25.4^\circ$	$0.334\angle 113^\circ$
$l = -1$ (CST)	$0.366\angle 41.0^\circ$	$0.379\angle$-50.1°	$0.370\angle$-139°	$0.362\angle 127^\circ$
(Meas.)	$0.285\angle$-150°	$0.335\angle 114^\circ$	$0.323\angle 18.0^\circ$	$0.317\angle$-87.0°

TABLE I

Simulated and measured transmission characteristics of microstrip OAM mode mux designed and fabricated on the Taconic TLY-5 substrate (ϵ_r = 2.2, thickness = 0.7874 $[mm]$), where f = 18 [GHz], input is $1\angle 0^\circ$, and A and ϕ in $A\angle\phi$ denote magnitude and phase, respectively.

IV. Supplementary Link Measurement for Different Separation Distance

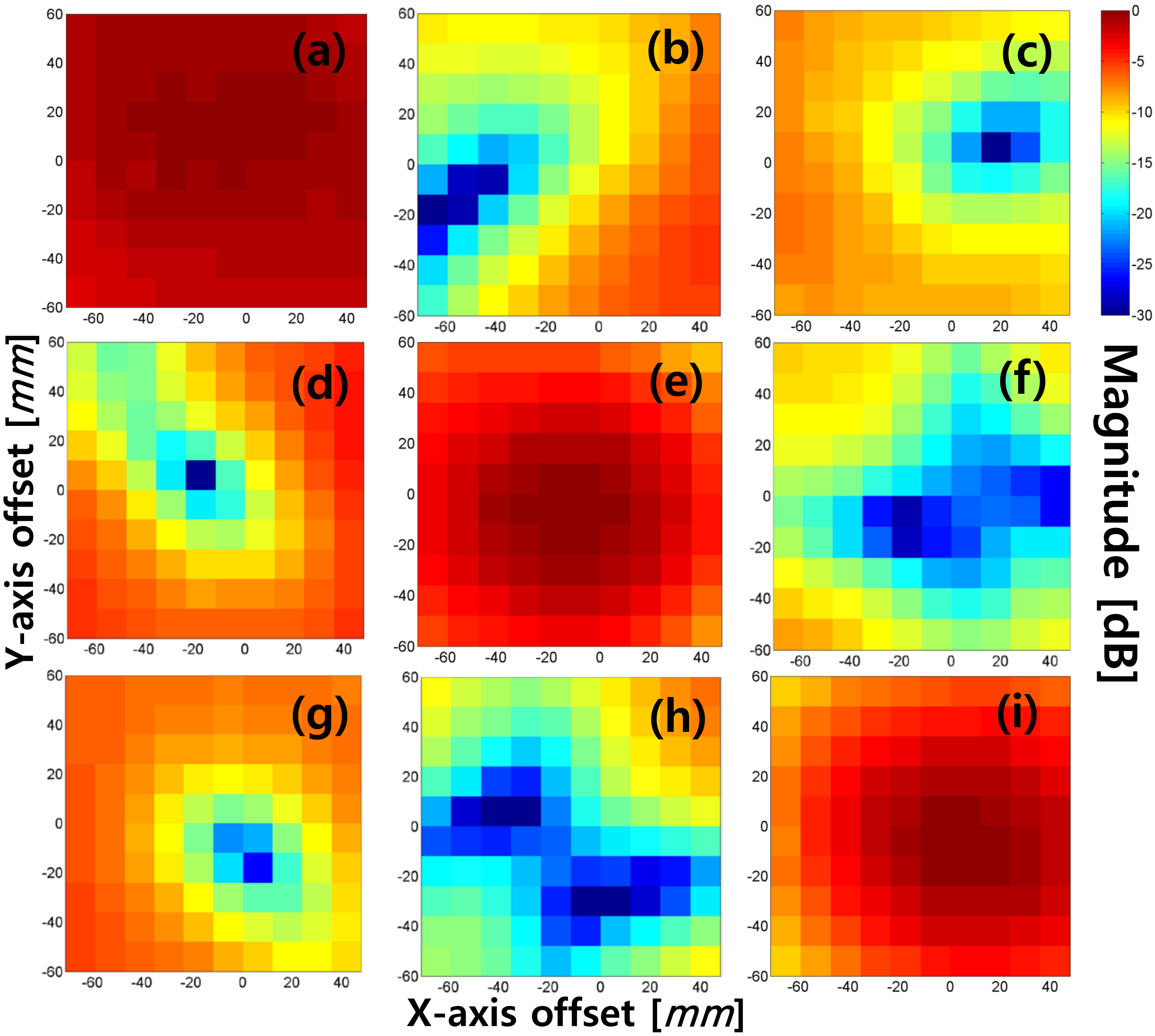

Fig. 4. Measured transmission coefficient (S_{21}) distributions for different transmitter (l_T) and receiver (l_R) OAM modes when the operating frequency is 18 [GHz] and the separation distance between the Tx and Rx is 1.5 [m]. During the measurement, l_T was fixed and l_R was manually chosen by connecting a spectrum analyzer to the corresponding OAM mode port of the OAM mode mux. **(a), (b), (c)** $l_R = 0$, $l_R = +1$, $l_R = -1$ for $l_T = 0$, respectively. **(d), (e), (f)** $l_R = 0$, $l_R = +1$, $l_R = -1$ for $l_T = +1$, respectively. **(g), (h), (i)** $l_R = 0$, $l_R = +1$, $l_R = -1$ for $l_T = -1$, respectively.

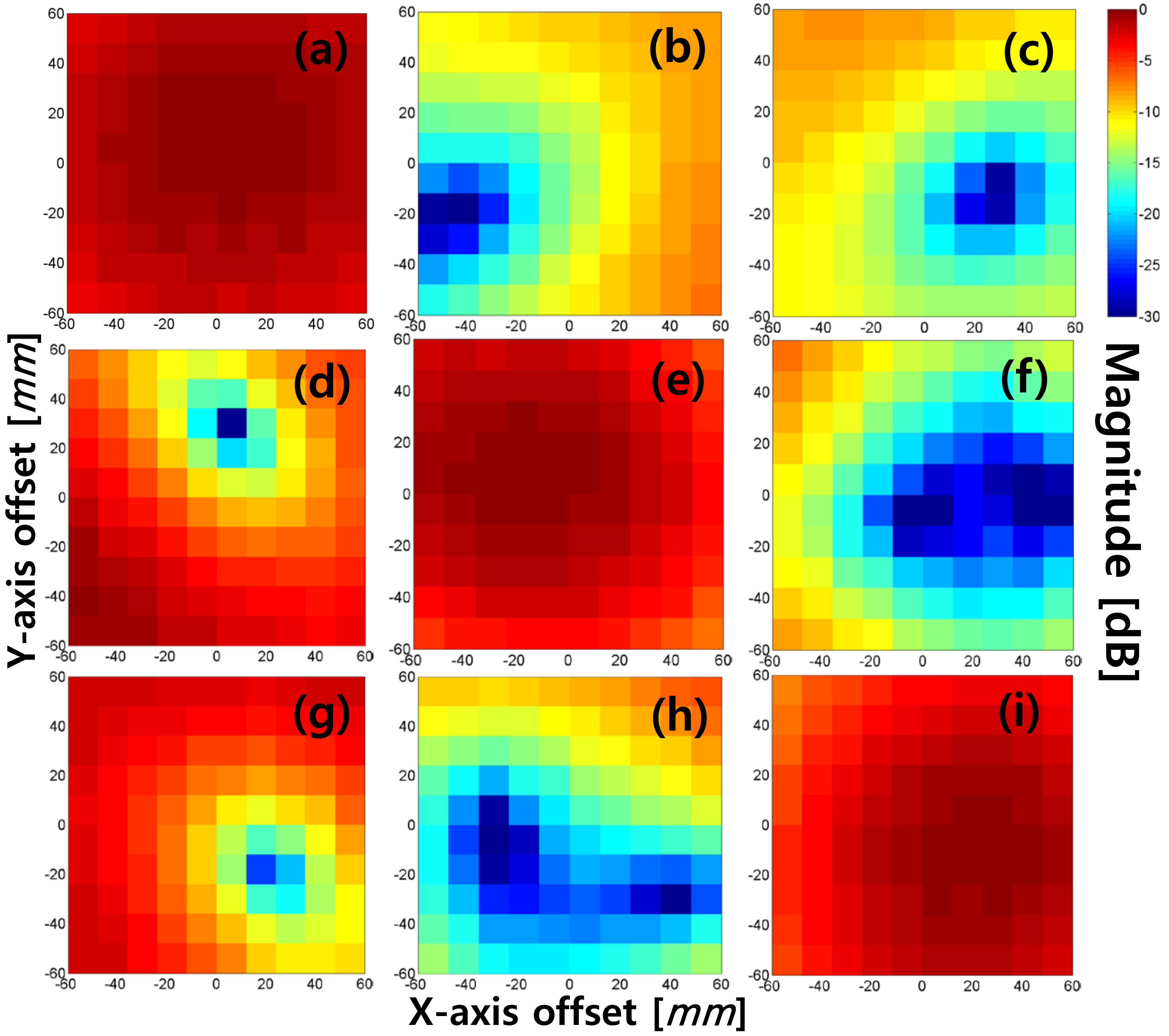

Fig. 5. Measured transmission coefficient (S_{21}) distributions for different transmitter (l_T) and receiver (l_R) OAM modes when the operating frequency is 18 [GHz] and the separation distance between the Tx and Rx is 2.5 [m]. During the measurement, l_T was fixed and l_R was manually chosen by connecting a spectrum analyzer to the corresponding OAM mode port of the OAM mode mux. **(a), (b), (c)** $l_R = 0$, $l_R = +1$, $l_R = -1$ for $l_T = 0$, respectively. **(d), (e), (f)** $l_R = 0$, $l_R = +1$, $l_R = -1$ for $l_T = +1$, respectively. **(g), (h), (i)** $l_R = 0$, $l_R = +1$, $l_R = -1$ for $l_T = -1$, respectively.

Tx S_{21}	Fixed Rx at 18 [GHz]		
	$l_R = 0$	$l_R = +1$	$l_R = -1$
$l_T = 0$	0	-16.3	-10.7
$l_T = +1$	-17.2	0	-12.7
$l_T = -1$	-6.83	-23.3	0
Total SIR	6.45	15.5	8.58

TABLE II

OPTIMAL TRANSMISSION COEFFICIENTS (S_{21}) AND TOTAL SIGNAL-TO-INTERFERENCE RATIO (SIR) WHEN OAM MODE NUMBER OF THE TRANSMITTER (TX) IS FIXED TO l_T, THE RECEIVER (RX) POSITION IS ANCHORED TO $(x_0, y_0) = (0, -24)$ [*mm*], AND THE SEPARATION DISTANCE BETWEEN THE TX AND RX IS 1.5 [m].

Tx S_{21}	Compensated Rx at 18 [GHz]			(x_0, y_0) [*mm*]
	$l_R = 0$	$l_R = +1$	$l_R = -1$	
$l_T = 0$	0	-16.2	-13	$(-12, -12)$
$l_T = +1$	-19.5	0	-28.8	$(-24, -12)$
$l_T = -1$	-14.7	-22.8	0	$(-24, -12)$
Total SIR	13.5	15.3	12.9	

TABLE III

BEST S_{21} AND TOTAL SIR WHEN THE RX POSITION FOR A FIXED l_T IS MOVED SLIGHTLY TO (x_0, y_0). THE OTHER PARAMETERS ARE THE SAME AS THOSE IN TABLE II.

Tx S_{21}	Fixed Rx at 18 [GHz]		
	$l_R = 0$	$l_R = +1$	$l_R = -1$
$l_T = 0$	0	-17	-12.8
$l_T = +1$	-7.33	0	-20
$l_T = -1$	-4.5	-20.8	0
Total SIR	2.68	15.5	12

TABLE IV

OPTIMAL TRANSMISSION COEFFICIENTS (S_{21}) AND TOTAL SIGNAL-TO-INTERFERENCE RATIO (SIR) WHEN OAM MODE NUMBER OF THE TRANSMITTER (TX) IS FIXED TO l_T, THE RECEIVER (RX) POSITION IS ANCHORED TO $(x_0, y_0) = (0, -24)$ [*mm*], AND THE SEPARATION DISTANCE BETWEEN THE TX AND RX IS 2.5 [m].

Tx S_{21}	Compensated Rx at 18 [GHz]			(x_0, y_0) [*mm*]
	$l_R = 0$	$l_R = +1$	$l_R = -1$	
$l_T = 0$	0	-15.3	-15.2	$(-12, -12)$
$l_T = +1$	-19.5	0	-22.3	$(-12, 0)$
$l_T = -1$	-24.2	-21.5	0	$(-24, 12)$
Total SIR	18.2	14.4	14.4	

TABLE V

BEST S_{21} AND TOTAL SIR WHEN THE RX POSITION FOR A FIXED l_T IS MOVED SLIGHTLY TO (x_0, y_0). THE OTHER PARAMETERS ARE THE SAME AS THOSE IN TABLE IV.

V. SUPPLEMENTARY MEASUREMENT OF 2×2 OERW MATRIX FEED

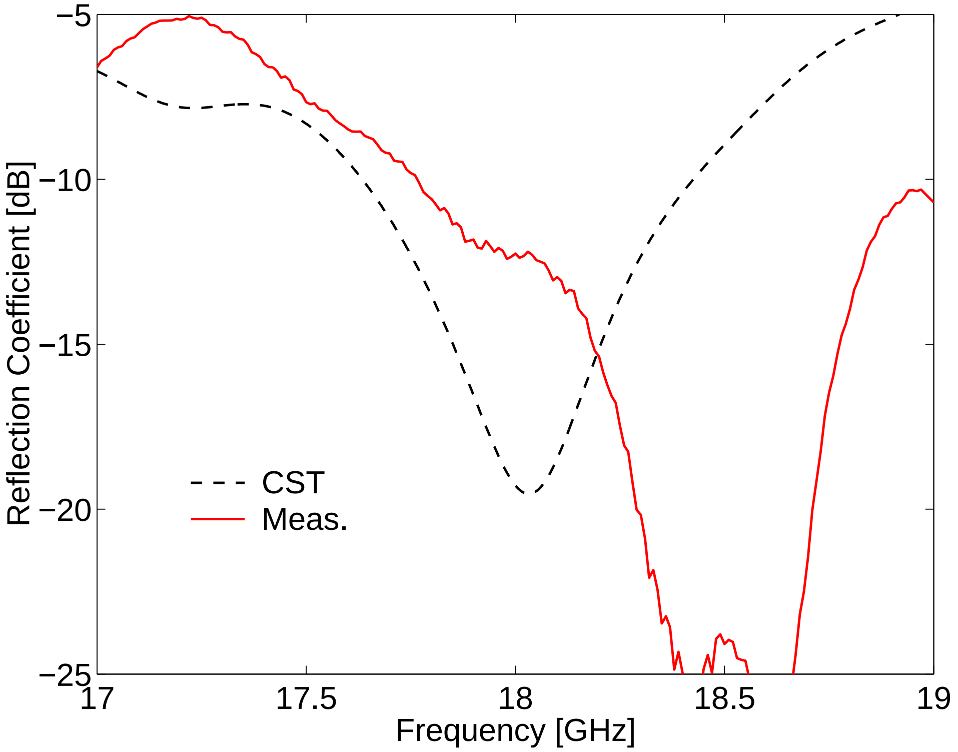

Fig. 6. Reflection coefficient of a 2×2 OERW matrix feed with $l = 0$ mode excitation with the same parameters in Fig. 2, the Taconic TLY-5 substrate (ϵ_r = 2.2, thickness = 0.7874 $[mm]$), patch dimensions = 7.48×4.69 $[mm^2]$, and coaxial feeding.

REFERENCES

[1] A. D. Yaghjian, "Approximate formulas for the far field and gain of open-ended rectangular waveguide," *IEEE Trans. Antennas Propagat.*, vol. 32, no. 4, pp. 378-384, April 1984.

[2] A. C. Ludwig, "The definition of cross polarization," *IEEE Trans. Antennas Propagat.*, vol. 21, no. 1, pp.116-119, Jan. 1973.

[3] G. Strang, *Linear Algebra and Its Applications,* 3rd Ed., Brooks/Cole, 1988.
